# Role of Non-coding RNAs in the Pathogenesis of Endometriosis

**DOI:** 10.3389/fonc.2020.01370

**Published:** 2020-08-04

**Authors:** Soudeh Ghafouri-Fard, Hamed Shoorei, Mohammad Taheri

**Affiliations:** ^1^Department of Medical Genetics, Shahid Beheshti University of Medical Sciences, Tehran, Iran; ^2^Department of Anatomical Sciences, Faculty of Medicine, Birjand University of Medical Sciences, Birjand, Iran; ^3^Urogenital Stem Cell Research Center, Shahid Beheshti University of Medical Sciences, Tehran, Iran

**Keywords:** miRNA, lncRNA, endometriosis, non-coding RNA, inflammation

## Abstract

Endometriosis is a disorder characterized by the presence of endometrial glands and stroma like lesions outside of the uterus. Although several hypothesis have tried to explain the underlying cause of endometriosis, yet the main cause remained obscure. Recent studies have shown contribution of non-coding RNAs in the pathogenesis of endometriosis. Two classes of these transcripts namely long non-coding RNAs (lncRNAs) and microRNAs (miRNAs) have mostly attracted attention of researchers. Several studies have reported aberrant expression of these transcripts in affected tissues from patients as well as animal models. Modulation of important signaling pathways such as PI3K/AKT, P38-MAPK, ERK1/2-MAPK and Wnt-β catenin by miRNAs and lncRNAs have potentiated these molecules as biomarkers or therapeutic agents in endometriosis. Single nucleotide polymorphisms with miR-126, miR-143 and miR-146b have been associated with risk of endometriosis. Moreover, miRNAs and lncRNAs control inflammatory responses, cell proliferation, angiogenesis and tissue remodeling, thus understanding the role of these transcripts in endometriosis is a possible way to develop novel diagnostic tests and therapeutic targets for this disorder.

## Introduction

Endometriosis is a condition that endometrial glands and stroma like lesions are detected in organs outside of the uterus (1). These lesions can involve the peritoneum or being presented as superficial implants or cysts on the ovary, or deep infiltrating lesions (2). Although the main etiology of endometriosis is not clear, numerous hypotheses have tried to explain the development of this disorder. Among the most appreciated hypotheses is the retrograde menstruation which can be accompanied by possible hematogenous or lymphatic circulation, thus leading to seeding of endometrial tissue in ectopic places. Yet, this phenomenon is much more prevalent than the occurrence of endometriosis. Hence, other hormonal or immune-related factors contribute in implantation and persistence of lesions in the pelvic cavity (3). Imperfect differentiation or migration of Müllerian residues during fetal period or transdifferentiate of circulating blood cells are other popular hypotheses regarding development of endometriosis (3). Notably, several genomics studies have shown remarkable alterations in gene profile in endometriosis (4). The genetics basis of this condition is complex and has not been explored yet, though, most studies have reported a polygenic/multifactorial mode for its inheritance (4). Most recently, non-coding RNAs have been demonstrated to contribute in the pathogenesis of endometriosis (5). These transcripts have regulatory roles on expression of protein-coding genes, thus regulate several signaling pathways. They are classified into two main classes according to their length: long non-coding RNAs (lncRNAs) with sizes more than 200 nucleotides and microRNAs (miRNAs) with sizes about 20 nucleotides. LncRNAs can regulate the genetic information flow, through modulating chromatin structure, transcription, splicing, mRNA stability, mRNA accesibility, and post-translational alterations. They have interaction domains for DNA, mRNAs, miRNAs, and proteins which are specified by nucleotide sequence and secondary structure (6). NONCODE database has indicated the presence of at least 100,000 lncRNAs in the human genome (7) which significantly surpluses the number of protein coding genes. There are complex interaction networks between lncRNAs and miRNAs. While certain miRNAs can regulate the stability and half-life of lncRNA, lncRNAs can compete with miRNAs for binding with the mRNA target sites (6). Being mostly located in the cytoplasm, miRNAs constitute critical regulators of gene expression. They mostly exert their effects at post-transcriptional level through binding with their targets and subsequent mRNA degradation and/or translational repression. In addition, miRNAs have been shown to exert specific nuclear functions being emphasized by the miRNA-guided transcriptional regulation of gene expression (8). The regulatory roles of miRNAs and lncRNAs in the expression of genes indicate their participation on the pathogenesis of human disorders. In the current study, we summarize the role of these transcripts in the pathophysiology of endometriosis.

## MiRNAs and Endometriosis

Several studies have reported aberrant expression of miRNAs in affected tissues or peripheral blood samples of patients. Zhang et al. have extracted exosomes from the serum of patients with endometriosis and healthy subjects, then assessed expression miRNAs by miRNA microarrays. They reported differential expression of 24 miRNAs between these two sets of samples. As confirmed by qPCR, expression of miR-22-3p and miR-320a was increased in serum exosomes of patients compared with controls (9). Another study has shown that exosomal miR-22-3p isolated from peritoneal macrophages increases proliferation, migration, and invasion of ectopic endometrial stromal cells via modulation of the SIRT1/NF-κB signaling pathway (10). Others have assessed expression profile of miRNAs peritoneal or tissue samples obtained from these patients. For instance, Zhou et al. have used miRNA microarray technique to identify miRNA signature in the ectopic endometrium samples. They reported over-expression of miR-3154 and miR-3926 in theses tissues compared with normal endometrium (11). Zhang et al. have isolated mononuclear cells from peritoneal fluid of patients with endometriosis and assessed expression of miRNAs in the supernatant of peritoneal fluid. They also purified human endometrial stromal cells from both endometrial and endometriotic tissues of these patients. They reported up-regulation of miR-146b peritoneal fluid supernatant and CD14 + monocytes/Macrophages of peritoneal fluid in endometriosis patients. This miRNA could inhibit the M1 polarization of endometrial stromal cells co-cultured macrophages (12). Table 1 shows the list of up-regulated miRNAs in samples obtained from patients with endometriosis.

**Table 1:** Up-regulated miRNAs in endometriosis.

**MicroRNA**	**Species**	**Numbers of clinical samples**	**Assessed cell line**	**Targets/Regulators**	**Signaling Pathways**	**Function**	**References**
miR-197-5p, miR-22-3p, miR-320a, miR-320b, miR-3692-5p, miR-4476, miR-4530, miR-4532, miR-4721, miR-4758-5p, miR-494-3p, miR-6126, miR-6734-5p, miR-6776-5p, miR-6780b-5p, miR-6785-5p, miR-6791-5p, miR-939-5p	Human	Isolated-exosome from serum samples of endometriosis patients (*n* = 20) and normal controls (*n* = 20)	–	–	–	Mentioned-microRNAs could be considered as potential biomarkers for endometriosis diagnosis.	(9)
miR-22-3p	Human	Peritoneal fluid samples from endometriosis patients (*n* = 20) and normal controls (*n* = 20)	HESCs	SIRT1	NF-κB	Exosomal miR-22-3p derived from pMϕ by regulating the SIRT1/NF-κB pathway could promote proliferation, migration, and invasion of human ectopic endometrial stromal cells (eESCs).	(10)
miR-92a	Human, mouse	Endometrial samples from women with progesterone-resistant endometriosis (*n* = 12) and with progesterone-responsive endometriosis (*n* = 11)	SHT290	PTEN	AKT	The expression of miR-92a is increased in progesterone resistant endometriosis samples. miR-92a via targeting PTEN/AKT signaling pathway could promote progesterone resistance in endometriosis.	(13)
miR-125b, miR-150, miR-342, miR-451a,	Human	Serum samples from endometriosis patients (*n* = 41) and normal controls (*n* = 59)	–	–	–	Mentioned-microRNAs could be considered as potential biomarkers for endometriosis diagnosis.	(13)
miR-342-3p	Human, mouse	Fat specimens from endometriosis patients (*n* = 10) and normal controls (*n* = 10)	Primary adipocyte cells	Cebpa, Cebpb, Ppar-γ, leptin, adiponectin, IL-6, HSL	–	miR-342-3p could affect the expression of metabolic genes in adipocytes of women with endometriosis. Therefore, it has a direct effect on fat metabolism.	(14)
miR-3154	Human, mouse	Ectopic endometrial tissues and serum from endometriosis patients (*n* = 68) and normal controls (*n* = 23)	EC109, EC520, EN211, EN307	–	–	This miRNA could be considered as a potential biomarker for endometriosis diagnosis.	(11)
miR-3926	Human, mouse	Ectopic endometrial tissues and serum from endometriosis patients (*n* = 68) and normal controls (*n* = 23)	EC109, EC520, EN211, EN307	–	–	This miRNA could be considered as a potential biomarker for endometriosis diagnosis.	(11)
miR-146b	Human	Peritoneal fluid samples from endometriosis patients (*n* = 74) and normal controls (*n* = 23)	ESCs, THP-1, PBMC	IRF5/IL-12p40	NF-kB	miR-146b via IRF5/IL-12p40/NF-kB axis is involved in the negative regulation of inflammation.	(12)
miR-33b	Rat	–	ESCs	ZEB1	Wnt/β-catenin	Overexpression of miR-33b via inhibiting ZEB1/Wnt/β-catenin signaling pathway could promote endometriosis.	(15)
miR-142-5p, miR-146a-5p, miR-1281, miR-940, miR-4634	Human	Eutopic endometrium samples from endometriosis patients (*n* = 38), normal controls (*n* = 38)	–	–	–	These miRs could be considered as potential biomarkers for endometriosis diagnosis.	(16)
miR-210-3p	Human, mouse	Normal endometria (*n* = 27), eutopic endometria (*n* = 57), ectopic lesions (*n* = 57)	HESCs, hEM15A, ISK, 293T	BARD1	–	Knockdown of miR-210-3p could induce a G2/M arrest of Ishikawa cells and ESCs under hypoxia. Therefore, miR-210-3p by targeting BARD1 could protect endometriotic cells from oxidative stress-induced cell cycle arrest.	(17)
miR-17-5p	Human	51 endometriosis patients and 51 controls	Endometrial tissue	–	–	There is a positive relationship between intrauterine bacterial colonization and increased levels of miR-17-5p. Therefore, it could be a biomarker of endometriosis.	(18)
miR-200b	Human	3 endometriotic and 3 nonendometriotic eutopic endometrium	Endometriotic mesenchymal stem cells (EMSCs)	–	–	In endometriosis, regulation of miR-200b may have a role in the modulating proliferation and differentiation of stem cells.	(19)
miR-451a, miR-486-5p, miR-130-3p	Human	Endometriosis patients (*n* = 54) and normal controls (*n* = 13)	–	–	–	Exosomal microRNAs could be involved in the progression of endometriosis.	(20)
miR-150-5p, miR-451a	Baboon	–	–	–	–	In the baboon model of endometriosis, the expression of these miRNAs is increased in response to simvastatin treatment. Therefore, it could be considered as a potential biomarker for endometriosis diagnosis.	(21)
miR-451a	Mouse	–	–	YWHAZ, CAB39, MAPK1, β-catenin, IL-6	–	Inhibition of miR-451a could reduce the established-lesion in an animal model of endometriosis.	(22)
miR-27a-3p, miR-451a, miR-144-5p, miR-1266-5p, miR-200c-3p, miR-200a-3p, miR-20b-5p, miR-200a-5p, miR-96-5p	Human	Endometrium (*n* = 6), endometriotic lesions (*n* = 6), PF (*n* = 6), and plasma (*n* = 6) from endometriosis patients	12Z, EEC-1, HUVEC	–	–	Mentioned-microRNAs could be considered as potential biomarkers for endometriosis diagnosis.	(23)
miR-122, miR-199a	Human	Endometriosis patients (*n* = 45) and normal controls (*n* = 35)	–	IL-6	–	Mentioned-microRNAs could be considered as biomarkers for the diagnosis of endometriosis.	(24)
miR-145	Human	Plasma samples of 55 patients with endometriosis and 23 normal controls	–	–	–	The mentioned-miR could be considered as a biomarker for the diagnosis of endometriosis.	(25)
miR-126, miR-145	Human	47 infertile patients with endometriosis, 47 normal controls	–	–	–	Overexpression of miR-126 and miR-145 in the mid-luteal phase of patients with endometriosis could play an important role in infertility due to endometriosis.	(26)
miR-106b-3p, miR-451a, miR-486-5p	Human	Peritoneal fluid (PF) samples form endometriosis patients (*n* = 60), normal control (*n* = 60)	–	–	–	Mentioned-microRNAs could be considered as a biomarker for the diagnosis of endometriosis.	(27)
miR-106b-3p, miR-130a-3p, miR-150-5p, miR-451a, miR-486-5p	Human	Endometriosis patients in the menstrual phase of the cycle (*n* = 12), normal control in the menstrual phase of the cycle (*n* = 4)	–	–	–	Analysis of microRNAs according to the phase of the menstrual cycle could be useful for the diagnosis of endometriosis.	(27)
miR-29c-3p, miR-185-5p, miR-195-5p	Human	Peritoneal fluid (PF) samples. Endometriosis samples (*n* = 126), 45 normal controls. (Based on the menstrual phase)	–	–	–	Analysis of microRNAs according to the phase of the menstrual cycle could be useful for the diagnosis of endometriosis.	(27)
miR-194-3p	Human	Midsecretory phase of the eutopic endometrium of women with endometriosis (*n* = 19), normal controls (*n* = 14)	HESCs	Progesterone receptor	–	In eutopic endometrium from women with endometriosis, mir-194-3p could repress the progesterone receptor and decidualization.	(28)
miR-33a-5p	Human	Plasma samples of endometriosis patients (*n* = 51), normal controls (*n* = 41)	–	–	–	The mentioned-miRNAs could be considered as a biomarker for the diagnosis of endometriosis	(29)
miR-139-5p, miR-139-3p, miR-202-5p, miR-506-3p, miR-150-5p, miR-202-3, miR-150-3p, miR-513c-5p, miR-193a-5p, miR-584-5p, miR-371a-5p, miR-216b-5p	Human	Paired samples of endometriomas (*n* = 4) and eutopic endometrium (*n* = 4)	HESCs, ST-T1b	HOXA9, HOXA10 for miR-139-5p	–	miR-139-5p by regulating HOXA9 and HOXA10 genes could be involved in endometriosis-associated infertility	(30)
miR-615-3p	Human	60 tissue samples (30 paired EC and EU) from patients with endometriosis (*n* = 30)	–	–	–	The mentioned-miR could be considered as a biomarker for the diagnosis of endometriosis	(31)
miR-29c, miR-200a, miR-145	Human	Tissues of 56 female patients with endometriosis, 38 normal controls	–	HOXA-10, HOXA-11, integrin αvβ3, IGFBP-1, CD44V6, N-cadherin, FAK	–	Mentioned-microRNAs via targeting several pathways could influence the endometrial receptivity in infertile patients with endometriosis	(32)
miR-29c	Human, baboon	Tissues: ectopic (*n* = 15) and normal controls (*n* = 11)	HuFs, HESCs,	FKBP4	–	miR-29c via targeting FKBP4 could modulate progesterone resistance in endometriosis	(33)
miR-451a	Human, baboon	41 endometriosis, 40 without visible signs of endometriosis	–	–	–	The level of miR-451a in serum is positively correlated with endometriotic lesion content.	(34)
miR-325, miR-492, miR-520e, miR-203a-3p, miR-93	Human	28 ovarian cancer samples, 17 normal samples, 33 endometriosis samples and	–	–	–	Mentioned-microRNAs could be considered as biomarkers for the diagnosis of endometriosis.	(35)
miR-27b-3p	Human	21 patients with endometriosis and 15 normal controls	HESCs, ISK	Ki-67, col-1, CTGF, fibronectin, TGF-β1, MMP2, MMP9	–	miR-27b-3p is upregulated in patients with endometriosis. Ginsenoside Rg3 extract (Rg3E) by modulating miR-27b-3p could decreases fibrotic and invasive nature of endometriosis	(36)
miR-29a, miR-148a, miR-100, let-7g	Human	Ectopic endometriotic tissues (*n* = 4), eutopic control endometrium	ISK, HESCs	BCL2, DNMT3B, OPRM1, Mip1α	–	The redox-sensitive microRNAs could be useful in the treatment of endometriosis-associated pain	(37)
miR-1304-3p, miR-544b, miR-3684, miR-494-5p, miR-4683, miR-6747-3p	Human	Eight patients with endometriosis and six normal controls	–	–	–	Mentioned-microRNAs could be used as a diagnostic biomarkers for endometriosis	(38)
miR-16-5p, miR-106b-5p, miR-145-5p	Human	Endometriotic tissues (*n* = 32), normal controls (*n* = 19)	–	EGFR2, PTEN, CXCR4	–	Mentioned-microRNAs could be used as a diagnostic biomarkers for endometriosis	(39)
miR-210	Human	Ectopic endometrial tissues (*n* = 10), eutopic endometrial tissues (*n* = 10)	CRL-7566	HIF-1α, Bcl-2, Beclin-1	–	The hypoxia-induced higher miR-210 expression through promoting autophagy and enhancing cell survival by Bcl2/Beclin-1 axis could contribute to pathological development of endometriosis	(40)
miR-122	Human	25 healthy women, 25 endometriosis patients	–	MCP-1, TGF-β1	–	Serum miR-122 could be useful in the evaluation of patients with endometriosis	(41)
miR-125b-5p, miR-150–5p, miR-342-3p, miR-143-3p, miR-145-5p, miR-500a-3p, miR-451a, miR-18a-5p	Human	24 endometriosis patients, 24 normal controls	–	–	–	Serum microRNAs could be considered as diagnostic markers of endometriosis	(42)

Several miRNAs have been shown to be down-regulated during the pathogenic process of endometriosis. Rekker et al. have used fluorescence-activated cell sorting to endometrial stromal cells from paired endometrial and endometrioma biopsies. Subsequently, they profiled miRNAs in endometriotic stroma using high-throughput sequencing method. They reported downregulation of miR-375 in these cells compared to eutopic cells. This miRNA has been shown to regulate expression of the endothelin 1 (EDN1) gene (30). Yang et al. have shown down-regulation of miR-200b, miR-15a-5p, miR-19b-1-5p, miR-146a-5p, and miR-200c while up-regulation of VEGFA in endometriotic tissues. They have speculated that the higher angiogenic and proteolytic activities observed in the eutopic endometrium could assist the implantation of these cells at ectopic regions (39). Table 2 summarizes the function and characteristics of miRNAs that are down-regulated in samples obtained from patients with endometriosis.

**Table 2:** List of down-regulated miRNAs in endometriosis.

**microRNA**	**Species**	**Numbers of clinical samples**	**Assessed cell line**	**Targets/Regulators**	**Signaling Pathways**	**Function**	**References**
miR-141	Human	Endometriotic tissue samples (*n* = 32), normal controls (*n* = 17)	ISK	–	TGF-β1/SMAD2	miR-141 via inhibiting the TGF-β1/SMAD2 signaling pathway could inhibit TGF-β1-induced EMT in endometriosis.	(9)
miR-3613-5p, miR-6755-3p	Human	24 endometriosis patients, 24 normal controls	–	–	–	Serum microRNAs could be considered as diagnostic markers of endometriosis.	(42)
miR-200b	Human	Three endometriosis patients, three normal controls	12Z, ST-T1b, HESCs	ZEB1, ZEB2, KLF4	–	miR-200b by targeting ZEB1, ZEB2, and KLF4 could affect the proliferation, invasiveness, and stemness of endometriotic cells.	(43)
miR-15a-5p	Human	31 patients with endometriosis and 31 normal controls	HESCs	VEGFA	–	miRNA-15a-5p by regulating VEGFA in endometrial mesenchymal stem cells could contribute to the pathogenesis of endometriosis.	(44)
miR-503	Human	Endometriotic tissues were from patients with ovarian endometriotic cysts (*n* = 32), normal control (eutopic, *n* = 8)	endometriotic cyst stromal cells (ECSCs), NESCs	VEGF-A, cyclin D1, Bcl-2, Rho A	–	miR-503 via targeting key molecules could induce apoptosis and cell-cycle arrest and could inhibit cell proliferation and angiogenesis in endometriosis.	(45)
miR-200b, miR-15a-5p, miR-19b-1-5p, miR-146a-5p, miR-200c	Human	Endometriotic tissues (*n* = 32), normal controls (*n* = 19)	–	VEGF-A	–	Mentioned-microRNAs could be used as a diagnostic biomarkers for endometriosis.	(39)
miR-3935, miR-4427, miR-652-5p miR-205-5	Human	Eight patients with endometriosis and 6 normal controls	–	–	–	Mentioned-microRNAs could be used as a diagnostic biomarkers for endometriosis.	(38)
miR-34a-5p	Human	Tissues: eutopic endometrial (*n* = 10) and ectopic endometrial (*n* = 10)	hEnSCs	VEGF-A	–	Overexpression of miR-34a-5p via targeting VEGFA could suppress the proliferation of endometrial-derived stem cells (EnSCs).	(46)
Let-7b-5p, Let-7c-5p, Let-7e-5p	Mouse	–	–	–	–	The family of let-7 in the serum shows a dysregulation in endometriosis.	(47)
miR-548l	Human	Ectopic endometriotic tissues (*n* = 4), eutopic control endometrium	ISK, HESCs	–	–	The mentioned-redox-sensitive miR could be useful in the treatment of endometriosis-associated pain.	(37)
miR-200c	Human, rat	normal endometrial (*n* = 12) and ectopic endometrial (*n* = 27) tissues	HESCs	MALAT1, ZEB1, ZEB2	–	miR-200c by targeting MALAT1/ZEB1/ZEB2 could suppress endometriosis.	(48)
miR-33b	Human	Tissues of 20 female patients with endometriosis, 15 normal controls	Endometrial tissue	VEGF, MMP-9	–	miR-33b via mediating apoptosis and altering VEGF or MMP-9 expression could affect proliferation and apoptosis of endometrial cells.	(49)
miR-30c	Human	Patients with endometriosis (*n* = 20), normal endometrial tissues (*n* = 18)	HESCs	PAI-1	–	Overexpression of miR-30c by targeting PAI-1 could repress the invasion, migration, proliferation, and adhesion of HESCs.	(50)
miR-424-5p	Human	Patients with endometriosis (*n* = 26), normal endometrial tissues (*n* = 26)	CRL-7566	FGFR1	STAT3	miR-424-5p by negatively regulating FGFR1 through STAT3 signaling expression could promote apoptosis and inhibit proliferation in CRL-7566 cells.	(51)
miR-34c-5p, miR-106a-5p, miR-182-5p, miR-200a-3p, miR-449b-5p	Human	60 tissue samples (30 paired EC and EU) from patients with endometriosis (*n* = 30)	–	FOXC1, FOXO1, CEBPA	–	Mentioned-microRNAs could be considered as biomarkers for the diagnosis of endometriosis.	(31)
miR-105-5p, miR-141-3p, miR-375, miR-429, miR-675-3p, miR-767-5p, miR-873-5p, miR-1298-5p, miR-6507-5p,	Human	Paired samples of endometriomas (*n* = 6) and eutopic endometrium (*n* = 6)	HESCs, ST-T1b	EDN1 for miR-375	–	miR-375 by targeting EDN1 could be involved in the regulation of invasive growth and cell proliferation in endometriosis development.	(30)
miR-134-5p, miR-3141, miR-4499, miR-6088, miR-6165, miR-6728-5p	Human	Isolated-exosome from serum samples of endometriosis patients (*n* = 20) and normal controls (*n* = 20)	–	–	–	Mentioned-microRNAs could be considered as potential biomarkers for endometriosis diagnosis.	(10)
miR-138	Rat, mouse	–	HESCs, THP-1	VEGF	NF-κB,	miR-138 via the VEGF/NF-κB signaling pathway could induce inflammation and apoptosis in endometriosis.	(52)
miR-451	Human	Tissue samples from endometriosis patients (*n* = 40) and normal controls (*n* = 20)	HESCs	YWHAZ, OSR1, TTN, CDKN2D	–	Downregulation of miR-451 could contribute to the pathogenesis of endometriosis by reducing apoptosis and promoting cell proliferation in the eutopic endometrium.	(53)
miR-543	Human	Eutopic endometrium samples from endometriosis patients (*n* = 38), normal controls (*n* = 38)	–	HOX10, ITGAV, ITGB3, OPN, ESR, PGR, CDH1, MMP	–	miR-543 is downregulated in patients with endometriosis and also is downregulated at the phase of implantation window. Therefore, it could affect embryo implantation in women with endometriosis-related infertility.	(16)
Let-7b, miR-6313	Human	Serum samples from endometriosis patients (*n* = 41) and normal controls (*n* = 59)	–	–	–	Mentioned-microRNAs could be considered as potential biomarkers for endometriosis diagnosis.	(13)
miR-202-3p	Human	Tissue samples from endometriosis patients (*n* = 27) and normal controls (*n* = 31)	HESCs	ROCK1	–	Suppression of miR-202-3p via targeting ROCK1 could enhance cell viability, invasion, and migration in ESCs.	(54)
miR-199a-5p	Human, rat	Control endometrial stromal cells (CSCs, *n* = 15), eESCs (*n* = 15)	HESCs, CSCs	ZEB1	PI3K/Akt/mTOR	miR-199a-5p via ZEB1/PI3K/Akt/mTOR signaling pathway could Inhibit the EMT of ovarian ectopic ESCs.	(55)
miR-20a	Human, mouse	Endometriosis patients (*n* = 60) and normal controls (*n* = 25)	PBMCs, NKCs, NK-92	ERG, HLX, perforin	STAT4	miR-20a via ERG/HLX/STAT4/perforin axis could mediate the cytotoxicity of natural killer (NK) cells in endometriosis.	(56)
Let-7b	Human, mouse	Fat specimens from endometriosis patients (*n* = 10) and normal controls (*n* = 10)	Primary adipocyte cells	Cebpa, Cebpb, Ppar-γ, leptin, adiponectin, IL-6, HSL	–	Let-7b could affect the expression of metabolic genes in adipocytes of women with endometriosis. Therefore, it has a direct effect on fat metabolism.	(14)
miR-205-5p	Human, mouse	Ectopic endometrial tissues and serum from endometriosis patients (*n* = 68) and normal controls (*n* = 23)	EC109, EC520, EN211, EN307	ANGPT2	ERK/AKT	miR-205-5p via ANGPT2/ ERK/AKT axis in endometrial stromal cells could inhibit human endometriosis progression.	(11)
miR-4497	Human, mouse	Ectopic endometrial tissues and serum from endometriosis patients (*n* = 68) and normal controls (*n* = 23)	EC109, EC520, EN211, EN307	–	–	This miR could be considered as a potential biomarker for endometriosis diagnosis.	(11)
miR-141-3p	Human	20 pairs of ectopic endometrial (EC) samples and eutopic endometrial (EU) samples, normal controls (*n* = 20)	HESCs	KLF-12	–	miR-141-3p via targeting KLF-12 could promote apoptosis and suppress cell proliferation and migration in ectopic ESCs.	(57)
miR-135a/b	Human	Samples of ectopic endometriosis lesions and eutopic endometrium tissue (*n* = 23)	–	–	–	Mentioned-microRNAs could be considered as potential biomarkers for endometriosis diagnosis.	(58)
miR-145, Let-7b	Human	3 endometriotic and 3 non-endometriotic eutopic endometrium	EMSCs	–	–	In endometriosis, regulation of miR-145 and let-7b may have a role in the modulating proliferation and differentiation of stem cells.	(19)
miR-451	Human, mouse	Endometriosis patients (*n* = 30) and normal controls (*n* = 30)	–	AXIN1, CDX2, CTNNB1	Wnt	miR-451 is downregulated in follicular fluid samples extracted from endometriosis patients. Downregulation of miR-451 by suppressing the Wnt signaling pathway in mouse and human oocytes could affect preimplantation embryogenesis.	(59)
miR-142-3p	Human	20 ectopic endometrial tissue samples, 20 eutopic endometrial tissues	CRL-7566, hEM15A, ECSCs, NESCs	KLF9	VEGFA	miR-142-3p by regulating KLF9-mediated autophagy could suppress endometriosis *in vitro* and *in vivo*.	(60)
miR-142-3p	Human	Serum samples from endometriosis patients (*n* = 41) and normal controls (*n* = 44)	12Z, ST-T1b, ECSCs	IL6ST, ITGAV, RAC1, WASL, ROCK2	STAT3	Downregulation of miR-142-3p via upregulating the expression of proinflammatory signaling receptors and cytoskeletal elements could promote the pathogenesis of endometriosis.	(61)
miR-375, miR-27a-3p, miR-30d-5p	Human	Endometrium (*n* = 6), endometriotic lesions (*n* = 6), PF (*n* = 6), and plasma (*n* = 6) from endometriosis patients	12Z, EEC-1, HUVEC	–	–	Mentioned-microRNAs could be considered as potential biomarkers for endometriosis diagnosis.	(23)
miR-488	Human, mouse	GSE5108 and GSE23339 chips	Endometrial tissues	FZD7	Wnt	Overexpression of miR-488 via inhibiting FZD7/Wnt pathway could reduce the proliferation, migration, and invasion of endometrial glandular epithelial cells.	(62)
miR-31	Human	Plasma samples of 55 patients with endometriosis and 23 normal controls	–	–	–	The mentioned-miR could be considered as a biomarker for the diagnosis of endometriosis.	(25)
miR-370-3p	Human	Sera and tissue from endometriosis patients (*n* = 20) and normal controls (*n* = 26)	HESCs	SF-1	–	miR-370-3p by regulating SF-1 could suppress proliferation in endometriotic cells.	(63)
miR-126-5p	Human	32 cases of ectopic endometrium and eutopic endometrium, 31 normal controls	EECs, ESCs, NESCs, 293T	BCAR3	–	Downregulation of miR-126-5p via negatively regulating BCAR3 could promote cell migration and invasion in endometriosis.	(64)
miR-3613-5p	Baboon	–	–	–	–	In the baboon model of endometriosis, the expression of miR-150-5p and miR-451a is decreased in response to simvastatin treatment. Therefore, they could be considered as potential biomarkers for endometriosis diagnosis.	(21)
miR-29c-3p, miR-1343-5p	Human	Peritoneal fluid (PF) samples form endometriosis patients (*n* = 60), normal control (*n* = 60)	–	–	–	Mentioned-microRNAs could be considered as potential biomarkers for endometriosis diagnosis.	(27)
miR-214	Human, mouse	Endometriosis patients (*n* = 24), normal control (*n* = 8)	Endometrial epithelial cells (EECs), HESCs	CTGF	–	miR-214-enriched exosomes could inhibit fibrogenesis in endometriosis.	(65)
miR-148a	Human	Endometriosis patients (*n* = 7), patients with endometriosis-associated ovarian cancer (EAOC, *n* = 7), normal controls (*n* = 6)	Hs 832(C).T	HLA-G, Caspase-3, Caspase-9, GPER	–	GPER/miR-148a/HLA-G signaling could mediate cell apoptosis in endometriosis.	(66)
miR-381	Human	Endometriosis patients (*n* = 6), patients with ovarian cancer (*n* = 3), normal control (*n* = 3)	TOV21G, TOV112D	PIK3CA	–	In endometriosis-associated clear cell and endometrioid ovarian cancer, miR-381 via targeting PIK3CA could regulate cell motility, growth, and colony formation.	(67)
miR-203	Human	Endometriosis patients (*n* = 6), patients with ovarian cancer (*n* = 3), normal control (*n* = 3)	TOV21G, TOV112D	–	–	The mentioned-miRNA could be considered as a biomarker for the diagnosis of endometriosis.	(67)
Let-7b	Mouse	–	–	ER-α, ER-ß, Cyp19a, KRAS, 4A, KRAS-4B, IL-6	–	Let-7b treatment of endometriosis could decrease inflammatory signaling (IL-6), decreased estrogen signaling (ER and Cyp19A1), and also decrease KRAS.	(68)
miR-21-5p, miR-181b-5p, miR-503-5p, miR-642a-3p, miR-3180-3p, miR-3180, miR-3937, miR-4498, miR-4690-5p, miR-6075, miR-6080, miR-6802-5p, miR-6820-5p, miR-7110-5p,	Human	Endometriosis patients (*n* = 16), normal control (*n* = 16)	HESCs	Caspase-3 for miR-21-5p	–	The extract of saponin via decreasing the expression miR-21-5p could induced apoptosis of endometrial cells in women with endometriosis.	(69)
miR-449b-3p	Human	Ectopic (endometrioma; *n* = 19), eutopic (*n* = 19), and normal (*n* = 35) endometrial tissues	HESCs, HUVECs	–	–	The aberrant expression of miR-449b-3p by effecting on endometrial stromal cell proliferation and angiogenesis could be involved in the development and progression of endometriosis.	(70)
miR-17	Human	Serum samples of endometriosis patients (*n* = 80), normal control (*n* = 60)	–	IL-4, IL-6	–	Investigating the expression of miR-17 could be considered as a noninvasive diagnostic test for the detection of endometriosis.	(71)
miR-154-5p, miR-196b-5p, miR-378a-3p	Human	Plasma samples of endometriosis patients (*n* = 51), normal controls (*n* = 41)	–	–	–	Mentioned-microRNAs could be considered as biomarkers for the diagnosis of endometriosis.	(29)

Diagnostic power of several miRNAs has been assessed in endometriosis. Maged et al. have shown that serum miR-122 and miR-199a had a sensitivity of 95.6 and 100.0% and a specificity of 91.4 and 100%, respectively, for diagnosis of disease status in women. Thus, these miRNAs are putative serum biomarkers for endometriosis (24). Moustafa et al. have shown up-regulation of miR-125b-5p, miR-150-5p, miR-342-3p, and miR-451a, while down-regulation of miR-3613-5p and let-7b in serum samples of patients with endometriosis compared with controls. The area under curve (AUC) values in receiver operating characteristic (ROC) curves ranged from 0.68 to 0.92 for these miRNAs. Notably, a classifier combining these miRNAs provided an AUC of 0.94 as verified in the independent set of individuals not included in the training set. Importantly, neither phase of menstrual cycle nor use of hormonal medicines affected the expression levels in these miRNAs. Thus, authors concluded the potential of the miRNAs panel in detection of endometriosis in clinical setting (13). Table 3 summarizes the results of studies which reported diagnostic value of miRNAs in endometriosis.

**Table 3:** Diagnostic value of miRNAs in endometriosis.

**Sample number**	**Area under curve**	**Sensitivity**	**Specificity**	**References**
Isolated-exosome from serum samples of endometriosis patients (*n* = 20) and normal controls (*n* = 20)	0.855 for miR-22-3p, 0.827 for miR-320a			(10)
Serum samples from endometriosis patients (*n* = 41) and normal controls (*n* = 59)	0.84 for miR-451a, 0.78 for Let-7b, 0.73 for miR-125b, 0.92 for miR-342, 0.76 for miR-3613	82.5% for Let-7b, 90% for miR-451a, 56.1% for miR-125b, 90% for miR-342, 92.7% for miR-3613	67.8% for Let-7b, 72.9% for miR-451a, 78% for miR-125b, 91.2% for miR-342, 61% for miR-3613	(13)
51 endometriosis patients and 51 controls		90%	76.5%	(18)
Serum samples from endometriosis patients (*n* = 45) and normal controls (*n* = 35)	0.963 for miR-122, 1.000 for miR-199a	95.6% for miR-122, 100.0% for miR-199a	91.4% for miR-122, 100.0% for miR-199a	(24)
Serum samples of endometriosis patients (*n* = 80), normal control (*n* = 60)	0.84			(71)
Plasma samples of endometriosis patients (*n* = 51), normal controls (*n* = 41)	0.72 for miR-154-5p	67% for miR-154-5p	68% for miR-154-5p	(29)
Patients with endometriosis (*n* = 41), individuals without visible signs of endometriosis (*n* = 40)	0.8599	85.37%	84.62%	(34)
Ovarian cancer samples (*n* = 28), normal samples (n17), endometriosis samples (*n* = 33)	0.775 for miR-492			(35)
Endometriosis patients (*n* = 24), normal controls (*n* = 24)	0.974 for miR-125b-5p, 0.808 for miR-150-5p, 0.760 for miR-342-3p, 0.926 for miR-143-3p, 0.901 for miR-500a-3p, 0.835 for miR-451a, 0.797 for miR-18a-5p, 0.718 for miR-6755-3p, 0.862 for miR-3613-5p	100% for miR-125b-5p	96% for miR-125b-5p	(42)

Few studies have reported association between single nucleotide polymorphisms (SNPs) within miRNA coding genes and risk of endometriosis. For instance, Sepahi et al. have genotyped the rs4636297 of miR-126 in 157 endometriosis patients and 252 healthy subjects. G allele of this SNP has been shown to protect against endometriosis. Moreover, significant association has also been detected between the A allele and severity of endometriosis (72). Zhang et al. have shown association between the CT/CC genotypes of miR-146b rs1536309 and the risk of pain symptom of endometriosis. Moreover, they detected lower levels of the miR-146b and higher pro-inflammatory functions in macrophages from CT/CC genotype carriers (12). Nimi-Hoveidi et al. have genotyped miR-143 rs41291957 and rs4705342 SNPs in infertile women with endometriosis and matched healthy subjects. They reported association between the C allele of rs4705342 and increased risk of endometriosis. In addition, the A allele of rs41291957 polymorphism was associated with susceptibility to endometriosis (73). Table 4 shows the results of studies which assessed association between miRNA SNPs and endometriosis.

**Table 4:** Association between polymorphisms with SNPs and risk of endometriosis.

**Number of cases and controls**	**Variant**	**References**
Endometriosis patients (*n* = 157) and healthy controls (*n* = 252)	miR-126 rs4636297 is associated with endometriosis risk and its severity. For ir-126 rs4636297 in allele (G vs. A) and genotype (GG vs. AA genotype), there was significant protection against endometriosis	(72)
Endometriosis patients (*n* = 74) and healthy controls (*n* = 23)	miR-146b rs1536309 C > T polymorphism is associated with the risk of pain symptom of endometriosis. rs1536309 CT/CC frequency is involved in increased pain susceptibility. miR-146b rs1536309 C > T polymorphism by regulating miR-146b expression was associated with the M1 polarization of macrophages	(12)
Infertile women (*n* = 77) with endometriosis and healthy controls (*n* = 226)	Among the groups of the study, there was a significant difference in the genotype distribution and allele frequency of miR-143 rs41291957 and miR-143 rs4705342 polymorphism. C allele and TC genotype were associated with an increased risk of endometriosis	(73)

## LncRNAs and Endometriosis

Expression levels of lncRNAs have been assessed in different samples obtained from patients with endometriosis or animal models of endometriosis. Cai et al. have assessed expression profiles of these transcripts in the uterus of rats with endometriosis and reported differential expression of a number of lncRNAs between endometriosis group and controls. They concluded that differentially expressed genes influence endometrial receptivity in rats with endometriosis during the implantation window which results in implantation failure (74). Using a high throughput method, Sun et al. have reported dysregulation of 948 lncRNA and 4,088 mRNA transcripts in ectopic endometrial tissue compared with paired eutopic endometrial tissue. These lncRNAs were mostly enriched in biological pathways associated with endometriosis, thus were thought to regulate expression of associated protein coding genes in cis- and/or trans (75). Huang et al. have assessed expression of the lncRNA UCA1 in ectopic and eutopic endometrium tissues of ovarian endometriosis patients and controls. They reported over-expression of this lncRNA in ectopic endometrium tissues compared with paired eutopic endometrium tissues in the majority of patients. They also demonstrated higher serum levels of this lncRNA after treatment. Notably, serum levels of UCA1 on the day of discharge were remarkably lower in patients with recurrence compared with patients without recurrence. Based on these results, authors concluded that UCA1 participates in the pathogenesis of ovarian endometriosis and may be a putative diagnostic and prognostic marker for this condition (76). Tables 5, 6 show up- and down-regulated lncRNAs in the endometriotic samples, respectively.

**Table 5:** Up-regulated lncRNAs in endometriosis.

**lncRNA**	**Species**	**Numbers of clinical samples**	**Assessed cell line**	**Targets/Regulators**	**Signaling Pathways**	**Function**	**References**
TC0101441	Human	10 pairs of ectopic and eutopic endometria from patients with ovarian endometriotic cysts, normal endometrium tissue (*n* = 10)	ECSCs	N-cadherin, snail, slug, TCF8/ZEB1	–	Extracellular vesicle-mediated transfer of the lncRNA-TC0101441 could enhance the migration and invasion of endometriosis	(77)
UCA1	Human	98 patients with endometriosis, 28 normal controls (serum samples)	–	–	–	LncRNA-UCA1 could be used as a diagnostic and prognostic biomarker for ovarian endometriosis	(76)
MALAT1	Human	Endometrial tissues from patients with endometriosis (*n* = 15), normal controls (*n* = 7)	Endometrial cells	MMP-9, caspase-3	NF-κB/iNOS	LncRNA-MALAT1 via NF-κB/iNOS pathway could facilitate endometrial cell apoptosis and also via targeting MMP-9 could suppress endometrial cell proliferation and invasion.	(78)
	Human	Paired eutopic and ectopic endometrium samples from patients with endometriotic (*n* = 30), normal controls (*n* = 30)	HESCs	HIF-1α, 3-MA, Beclin1	–	LncRNA-MALAT1 via targeting HIF-1α/3-MA/Beclin1 could mediate hypoxia-induced pro-survival autophagy of HESCs in endometriosis.	(79)
CCDC144NL-AS1	Human	Paired ectopic and eutopic endometria from patients with endometriotic (*n* = 34), normal controls (*n* = 27)	hEM15A, HUVECs	Vimentin, MMP-9	–	lncRNA-CCDC144NL-AS1 knockdown could decrease migration and invasion phenotypes in endometrial stromal cells from endometriosis	(80)
BANCR	Rat	–	–	VEGF, MMP-2, MMP-9	ERK/MAPK	lncRNA-BANCR inhibitor via inhibiting ERK/MAPK signaling pathway could repress the development of ectopic endometrial tissues	(81)
SNORD3A, TCONS_00006582	Human	Eutopic endometrium samples (*n* = 17), normal samples (*n* = 17)	–	–	–	lncRNAs could be considered as novel diagnostic biomarkers and therapeutic targets for endometriosis	(82)
NONRATT006252, gi|672027621|ref|XR_592747.1|, gi|672045999|ref|XR_591544.1|, gi|672066614|ref|XR_594547.1, NONRATT006252, gi|672045999|ref|XR_591544.1|	Rat	–	–	Dlx3, P2ry6, Adamts7	–	During the implantation window process, changes in the expression of lncRNAs could affect endometrial receptivity in rats with endometriosis.	(74)
AFAP1-AS1	Human, mouse	Paired eutopic and ectopic endometrium samples from patients with ovarian endometriotic cysts (*n* = 18), normal controls (*n* = 10)	HESCs, ISK	ZEB1, E-cadherin	–	lncRNA-AFAP1-AS1 by targeting ZEB1 could promote EMT of endometriosis. Knockdown of AFAP1-AS1 could inhibit the growth of endometrial epithelial cells through inhibiting E2-induced activity of promoter site pGL3-P886 of transcription factor ZTB1.	(83)
LINC01279, MSC-AS1	Human	GSE7305, GSE7846, GSE29981 and E-MTAB-694 datasets	–	–	–	LINC01279 and MSC AS1 could be associated with the pathogenesis of endometriosis.	(84)
CHL1-AS2	Human	Paired eutopic and ectopic endometrium samples from patients with endometriotic (*n* = 31), normal controls (*n* = 30)	HL-60, Jurkat	–	–	lncRNA-CHL1-AS2 could be involved in the endometriosis development.	(85)
H19	Human	Eutopic endometrial tissues from patients with endometriosis (*n* = 23), normal controls (*n* = 23)	HESCs, 293T	miR-216a-5p, ACTA2	–	The estrogen-regulated lncRNA-H19/ACTA2/miR-216a-5p axis could mediate the invasion and migration of eutopic endometrial stromal cells (euESCs) in women with endometriosis.	(78)
HOXA11-AS1	Human	Paired eutopic and ectopic endometrium samples from patients with endometriotic (*n* = 30), normal controls (*n* = 15)	–	HOXA9, HOXA10, HOXA11, HOXA13		Although lncRNA-HOXA11-AS1 had no role on endometrial receptivity in endometriosis-associated infertility, it could influence the development of peritoneal endometriosis.	(86)
AC068282.3, RP11-369C8.1, RP11-432J24.5, GBP1P1	Human	Eutopic endometrial tissues from patients with endometriosis (*n* = 40), normal controls (*n* = 28)	–	–	–	lncRNAs could be considered as novel diagnostic biomarkers and therapeutic targets for endometriosis.	(87)
CHL1-AS2, XLOC_009813, LOC643650, XLOC_009813, LOC255167, LOC400043, XLOC_012904, XLOC_l2_009510, AFAP1-AS1, XR_113107, XLOC_002900, LOC284576, XLOC_002900, XLOC_004907, XLOC_009813, XLOC_l2_008976, XLOC_006043, LOC100128893, XLOC_002900, XR_110229	Human	Paired eutopic and ectopic endometrium samples from patients with endometriotic (*n* = 25)	–	–	–	lncRNAs could be considered as novel diagnostic biomarkers and therapeutic targets for endometriosis.	(75)
PRKAR2B	Human	Paired eutopic and ectopic endometrium samples from patients with endometriotic (*n* = 3), normal controls (*n* = 3)	–	–	PI3K-Akt, NF-κB, TGF-β, MAPK	lncRNA could be considered as a novel diagnostic biomarker and therapeutic target for endometriosis.	(88)
NONHAT076754	Human	Paired eutopic and ectopic endometrium samples from patients with ovarian endometriotic cysts (*n* = 92)	HESCs	ZO-1, E-cadherin, N-cadherin	–	Exosomal lncRNA-NONHAT076754 could facilitate endometriosis invasion.	(89)
aHIF	Human	Ectopic (*n* = 30) and normal (*n* = 16) endometrium samples	ECSCs, HUVECs	VEGF-A, VEGF-D	–	Exosomal lncRNA-aHIF could Promote angiogenesis in endometriosis.	(90)

**Table 6:** Down-regulated lncRNAs in endometriosis.

**lncRNA**	**Species**	**Numbers of clinical samples**	**Assessed cell line**	**Targets/Regulators**	**Signaling Pathways**	**Function**	**References**
CLEC2D	Human	Paired eutopic and ectopic endometrium samples from patients with endometriotic (*n* = 3), normal controls (*n* = 3)	–	–	PI3K-Akt, NF-κB, TGF-β, MAPK	This lncRNA could be considered as a novel diagnostic biomarker and therapeutic target for endometriosis.	(88)
ABO, TCONS_08347373	Human	Eutopic endometrium samples (*n* = 17), normal samples (*n* = 17)	–	–	–	lncRNAs could be considered as novel diagnostic biomarkers and therapeutic targets for endometriosis.	(82)
MALAT1	Human	Granulosa cells (GCs) from endometriosis patients (*n* = 52) and controls (*n* = 52)—(follicles ≥10)	KGN	p21, CDK2, cyclin D1	ERK/MAPK	Downregulation of lncRNA-MALAT1 by upregulating p21 via activation of the ERK/MAPK pathway could inhibit granulosa cell proliferation in endometriosis.	(91)
H19	Human	Eutopic endometrial tissues from endometriosis patients (*n* = 10), normal controls (*n* = 10)	HESCs	Let-7, IGF1R	–	lncRNA-H19 via IGF signaling pathway could alter the growth of stromal cells in the endometrium of women with endometriosis.	(92)
	Human	Endometriosis patients (*n* = 20), controls (*n* = 16) (peritoneal fluid samples)	HESCs	miR-342-3p, IER3	–	The level of IL-17 and the percentage of Th17 cells/CD4+ T cells are decreased when lncRNA-H19 overexpressed. Therefore, upregulated-lncRNA-H19 through miR-342-3p/IER3 pathway could inhibit Th17 cell differentiation to relieve endometriosis.	(93)
NONRATT003997, gi|672033904|ref|XR_589853.1|	Rat	–	–	Dlx3, P2ry6, Adamts7	–	During the implantation window process, changes in the expression of lncRNAs could affect endometrial receptivity in rats with endometriosis.	(74)
SRA	Human	Endometriotic samples from women with endometriosis (*n* = 24), normal controls (*n* = 24)	HESCs	Estrogen receptor	–	Silencing of SRA1 via regulating ER expression could decrease stromal cells growth in ovarian endometriosis.	(94)
AC002454.1, RP11-403H13.1	Human	Eutopic endometrial tissues from patients with endometriosis (*n* = 40), normal controls (*n* = 28)	–	–	–	LncRNAs could be considered as novel diagnostic biomarkers and therapeutic targets for endometriosis.	(87)
LOC100505776, UCA1, LOC100506860, XLOC_012981, LINC00261, LOC100507043, LOC100507218, LOC440335, XLOC_l2_013295, LINC00116, MSX2P1, XLOC_l2_013295, KLKP1, XLOC_005677, XLOC_l2_013295, XLOC_001243, XLOC_003147, LOC100507043	Human	Paired eutopic and ectopic endometrium samples from patients with endometriotic (*n* = 25)	–	–	–	LncRNAs could be considered as novel diagnostic biomarkers and therapeutic targets for endometriosis.	(75)
LINC00261	–	–	CRL-7566	–	–	LINC00261 could inhibit endometriosis cell growth and migration.	(95)

**Figure 1:**
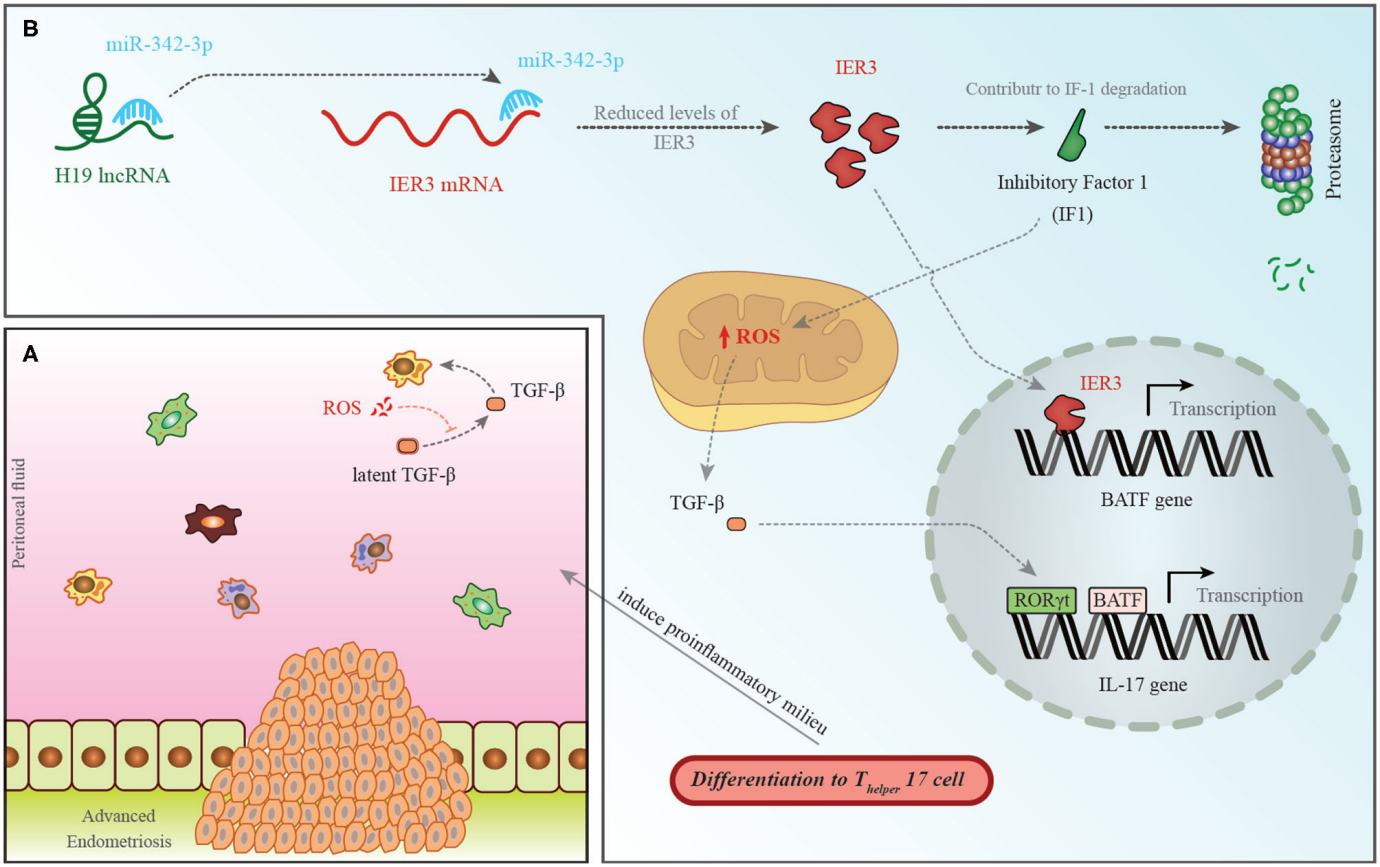
**(A)** A pro-endometriotic microenvironment produced by an existing endometriotic lesion provides the appropriate micro-environment for the progression of this disorder. After the buildup of cells by a previously established lesion, these elements show distinctive features that destroy immune surveillance (96). **(B)** H19 levels have been shown to be decreased in the PBMCs of patients with endometriosis. This is accompanied with an increase in miR-342-3p levels. This miRNA binds with the 3′ UTR of IER3, thus inhibiting its expression (93). IER3 participates in proteasomal degradation of IF-1. Decrease in the levels of IF-1 leads to increase in reactive oxygen species (ROS) levels (97). ROS increases active extracellular TGF-β levels. This cytokine influences RORγt, thus activating transcription of IL-17 and leading to differentiation of TH17 cells (98).

Among down-regulated lncRNAs is H19 whose role in the pathogenesis of endometriosis has been shown in Figure 1.

**Figure 2:**
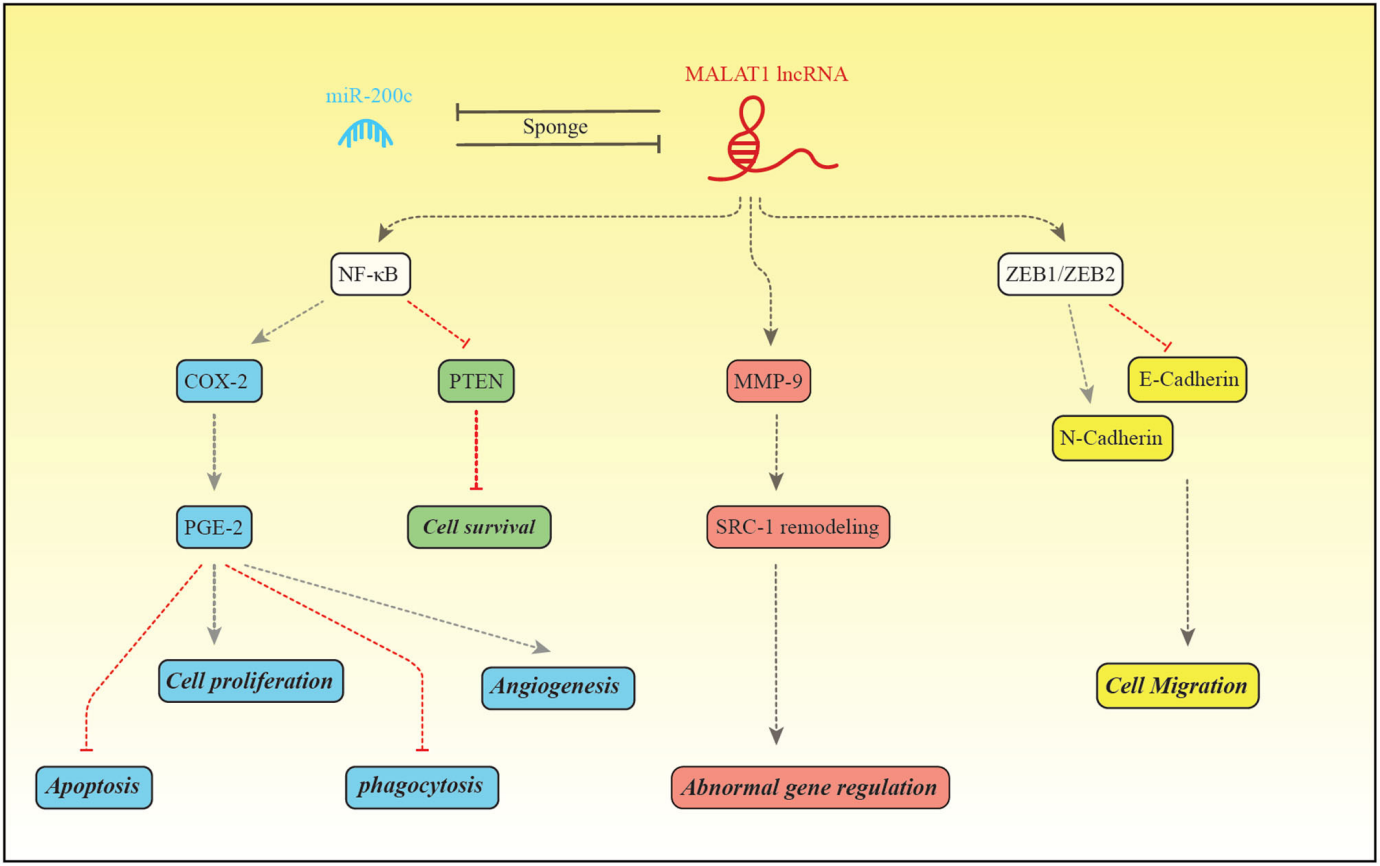
MALAT1 and miR-200c regulate expression of each other through the sponging mechanism. Liang et al. have reported down-regulation of MALAT1 and up-regulation of miR-200c in patients with endometriosis (48). MALAT1 increase expression of NF-κB which in turn binds with PGE-2 to enhance its expression (99). PGE-2 activates BCL2/BAX through interaction with EP2/EP4 receptor and suppresses intrinsic apoptotic pathway (100). PGE2 also activates cell proliferation through EP2/EP3 (101). In addition, PGE-2 suppresses MMP2, CD36 and annexin A2 in macrophages, thus inhibits phagocytic activity of macrophages. These effects facilitate implantation and growth of endometrial tissue in the peritoneum (101). PGE-2 influences angiogenic activity and cell cycle progression through increasing expression of VEGF and inhibiting PTEN, respectively (99). MALAT1 can enhance MMP9 levels. MMP9 increases production of the truncated isoform of Steroid receptor coactivator-1. MALAT1 also increases transcription of ZEB1/ZEB2, therefore induces mesenchymal cell phenotype which is accompanied by enhancement of cell migration (48).

The expression pattern of MALAT1 has been assessed in a number of studies among them is the study by Liang et al. that reported down-regulation of this lncRNA in the endometriosis (48). Figure 2 depicts the molecular mechanism of involvement of MALAT1 in this disorder.

## Interaction Between miRNAs and lncRNAs in the Pathogenesis of Endometriosis

Based on the significant roles of lncRNAs and miRNAs in the pathogenesis of endometriosis and the presence of functional interactions between these two sets of transcripts, it is expected that lncRNA/miRNA pairs could regulate certain aspects of endometriosis. LncRNAs can act as a competing endogenous RNA (ceRNA) for miRNAs to affect their bioavailability of these transcripts. Assessments in the endometrial tissues have led to identification of a number of miRNAs that are inhibited by the lncRNA H19. For instance, Ghazal et al. have shown that H19 serves as a molecular sponge to decrease the availability of let-7. This interaction leads to over-expression of the downstream target of let-7, IGF1R, thus increasing the proliferation of endometrial stroma cells. They also demonstrated down-regulation of H19 in the eutopic endometrium of patients with endometriosis and speculated that the subsequent decrease in the IGF1R activity might diminish endometrial stromal cell proliferation and negatively influence the endometrial receptivity for pregnancy (92). In addition, Xu et al. have demonstrated the role of the estrogen-modulated H19/ACTA2/miR-216a-5p axis in the regulation of invasion and migration of eutopic endometrial stromal cells in subjects with endometriosis (78). Liu et al. have reported the significance of H19/miR-342-3p/IER3 axis in suppression of Th17 cell differentiation and decreasing the risk of endometriosis (93). A recent high throughput study of RNA profile of the ectopic and eutopic endometrium of patients has led to construction of the ceRNA network. Assessment of the RNA interaction network in endometriosis has resulted to identification of the role of miRNAs and lncRNAs that are associated with cyclin-dependent kinase 1 (CDK1) and proliferating cell nuclear antigen (PCNA). These genes regulate the growth and apoptosis of endometrial stromal cells, thus are involved in the pathophysiology of endometriosis. Taken together, the RNA interactive network has critical role in this disorder (102).

## Discussion

Several studies have assessed expression profile of lncRNAs and miRNAs in tissues/blood samples obtained from patients with endometriosis. Association between genomic variants of miRNAs and endometriosis has also been another research avenue. However, the latter field has been less explored for lncRNAs. Considering the presence of myriads of SNPs within lncRNA coding genes that modulate their expression and regulatory functions on their targets, assessment of their association with the risk of endometriosis is a necessary step for identification of the role of these transcripts. Non-coding RNAs have fundamental roles in the development of endometriosis. Their role in this process has been highlighted not only by the studies which reported their aberrant expression in patients' samples, but also by the investigations which showed modulation of their expression by therapeutic agents. For instance, Quercetin (3,3′,4′,5,7-pentahydroxyflavone) as a phytochemical agent with antioxidant, anti-inflammatory and antiangiogenic characteristics has been shown to suppress the proliferation and induce cell cycle arrest in VK2/E6E7 and End1/E6E7 cells. Moreover, it has exerted antiproliferative and anti-inflammatory impacts on endometriosis autoimplanted mouse models. This effect has been accompanied by induction of miR-503-5p, miR-1283, miR-3714 and miR-6867-5p in both cell lines and stimulation of miR-503-5p and miR-546 expression in the animal model (103). Saponin extract, as another natural therapeutic agent has been shown to reduce expression of miR-21-5p in the human endometrial stromal cells from patients with endometriosis. Suppression of this miRNA could induce apoptosis in these cells. These results imply that he therapeutic effect of saponin is exerted through modulation of specific miRNAs (69).

Expressions of miRNAs have been assessed in different samples from patients with endometriosis such as endometrium, peripheral blood and peritoneal fluid. There are some cases of inconsistency between these studies. For instance, expression of miR-451a has been shown to be up-regulated in serum (13), exosomes (20) and endometriosis lesions of patients with endometriosis (23) as well as samples obtained from mouse models of endometriosis (22). However, another study has reported downregulation of miR-451 in the eutopic endometrial tissues of patients with endometriosis compared with control tissues (53). The lncRNA UCA1 has been reported to be up-regulated in ectopic endometrium tissues compared with paired eutopic endometrium tissues in the majority of patients using qRT-PCR (76). On the other hand, a microarray analysis showed down-regulated of this lncRNA in ovarian ectopic endometrial tissue compared with paired eutopic endometrial tissue (75). Similar discrepancy has been observed for MALAT1. While it has been upregulated in endometrial tissues from patients with endometriosis compared with normal controls (78), it was downregulated in granulosa cells from endometriosis patients compared with controls (91). The heterogeneity of samples and the method of expression analysis can partly explain the inconsistency of these results.

Mechanistically, lncRNAs can sponge miRNAs, regulate expression of inflammatory factors, alter cell proliferation, migration and apoptosis of endometrial cells. They might also affect implantation process (104, 105). Several transcription factors and signaling pathways have been regulated by lncRNAs in the endometrial tissues. Examples are HOX genes, N-cadherin, snail, slug, TCF8/ZEB1, matrix metalloproteinase, apoptosis related genes such as caspases and autophagy-related genes such as Beclin1.

The advent of next generation sequencing has enhanced the pace of identification of dysregulated non-coding RNAs in all human diseases including endometriosis. This technique has been applied by Khalaj et al. to identify signature of these transcripts in extracellular vesicles (EVs) obtained from endometriosis patient tissues and plasma samples compared with controls. Authors have demonstrated the presence of distinctive signatures of miRNAs and lncRNAs indicating their participation in the pathogenesis of endometriosis. Dysregulated transcripts were enriched in the pathways related to immune and metabolic functions. Their results indicated that endometriosis-associated EVs transport distinctive cargo and influence the disease course by modulation of inflammation, angiogenesis and proliferation (23). Moreover, exosomal miRNAs isolated from peritoneal macrophages have been shown to increase proliferation, migration, and invasion of ectopic endometrial stromal cells (10). Thus, these transcripts have fundamental roles in the pathogenesis of endometriosis. Taken together, these studies have opened a new research era for identification of the pathophysiology of endometriosis. Another technical development which has facilitated identification of this process has been the cell sorting technique. This technique has paved the way for cell-type-specific analysis of ectopic tissues to recognize the interactions between different cell types during the course of disease (30).

Considering the unavailability of affected tissues in the endometriosis except through invasive methods, identification of biomarkers in the serum of patients has a practical significance. Recent studies have demonstrated appropriate diagnostic power and sensitivity and specificity values for several miRNAs in this regard. Several miRNAs panels are expected to be applied in the clinical settings with high diagnostic power values. In spite of the presence of these supporting results, there is no consensus on a panel for the diagnosis of endometriosis, since most of studies have been conducted in small samples sizes of patients and their results have not been verified in independent samples. Besides, based on the differences in the source of controls, the applied techniques and the biological sources, meta-analysis of the obtained data is complicated. The diagnostic power of lncRNAs in the endometriosis has been less studied. Thus, future studies are needed to assess this aspect as well.

Taken together, based on the results of human and animal investigation, both miRNAs and lncRNAs participate in the pathogenesis of endometriosis. A more comprehensive assessment of these transcripts using the high throughput methods and identification of the functional links between these two sets of transcripts would facilitate identification of the pathogenesis of endometriosis and recognition of possible therapeutic targets in this regard.

## Author Contributions

HS performed the data collection. MT and SG-F wrote the draft and revised it. MT designed the hypothesis. All authors contributed to the article and approved the submitted version.

## Conflict of Interest

The authors declare that the research was conducted in the absence of any commercial or financial relationships that could be construed as a potential conflict of interest.

## References

[1] Giudice LC. Endometriosis. New Engl J Med. (2010) 362:2389–98. 10.1056/NEJMcp1000274 20573927 PMC3108065

[2] Nisolle M Donnez J. Peritoneal endometriosis, ovarian endometriosis, and adenomyotic nodules of the rectovaginal septum are three different entities. Fertil Steril. (1997) 68:585–96. 10.1016/S0015-0282(97)00191-X 9341595

[3] Parasar P Ozcan P Terry KL. Endometriosis: epidemiology, diagnosis and clinical management. Curr Obstet Gynecol Rep. (2017) 6:34–41. 10.1007/s13669-017-0187-1 29276652 PMC5737931

[4] Hansen KA Eyster KM. Genetics and genomics of endometriosis. Clin Obstet Gynecol. (2010) 53:403–12. 10.1097/GRF.0b013e3181db7ca1 20436317 PMC4346178

[5] Panir K Schjenken JE Robertson SA Hull ML. Non-coding RNAs in endometriosis: a narrative review. Human Reprod Update. (2018) 24:497–515. 10.1093/humupd/dmy014 29697794

[6] Fernandes JCR Acuña SM Aoki JI Floeter-Winter LM Muxel SM. Long non-coding RNAs in the regulation of gene expression: physiology and disease. Noncoding RNA. (2019) 5:17. 10.3390/ncrna5010017 30781588 PMC6468922

[7] Zhao Y Li H Fang S Kang Y Wu W Hao Y. NONCODE 2016: an informative and valuable data source of long non-coding RNAs. Nucleic Acids Res. (2016) 44(D1):D203–8. 10.1093/nar/gkv1252 26586799 PMC4702886

[8] Catalanotto C Cogoni C Zardo G. MicroRNA in control of gene expression: an overview of nuclear functions. Int J Mol Sci. (2016) 17:1712. 10.3390/ijms17101712 27754357 PMC5085744

[9] Zhang L Li H Yuan M Li D Sun C Wang G. Serum exosomal microRNAs as potential circulating biomarkers for endometriosis. Dis Mark. (2020) 2020:2456340. 10.1155/2020/2456340 32076458 PMC7008302

[10] Zhang L Li H Yuan M Li D Wang G. Exosomal miR-22-3p derived from peritoneal macrophages enhances proliferation, migration, and invasion of ectopic endometrial stromal cells through regulation of the SIRT1/NF-κB signaling pathway. Eur Rev Med Pharmacol Sci. (2020) 24:571–80. 10.26355/eurrev_202001_20033 32016958

[11] Zhou C-F Liu M-J Wang W Wu S Huang Y-X Chen G-B. miR-205-5p inhibits human endometriosis progression by targeting ANGPT2 in endometrial stromal cells. Stem Cell Res Ther. (2019) 10:287. 10.1186/s13287-019-1388-5 31547870 PMC6757391

[12] Zhang Z Li H Zhao Z Gao B Meng L Feng X. miR-146b level and variants is associated with endometriosis related macrophages phenotype and plays a pivotal role in the endometriotic pain symptom. Taiw J Obstetr Gynecol. (2019) 58:401–8. 10.1016/j.tjog.2018.12.003 31122533

[13] Moustafa S Burn M Mamillapalli R Nematian S Flores V Taylor HS. Accurate diagnosis of endometriosis using serum microRNAs. Am J Obstetr Gynecol. (2020). 10.1016/j.ajog.2020.02.050 32165186

[14] Zolbin MM Mamillapalli R Nematian SE Goetz L Taylor HS. Adipocyte alterations in endometriosis: reduced numbers of stem cells and microRNA induced alterations in adipocyte metabolic gene expression. Reprod Biol Endocrinol. (2019) 17:36. 10.1186/s12958-019-0480-0 30982470 PMC6463663

[15] Zhang H Li G Sheng X Zhang S. Upregulation of miR-33b promotes endometriosis via inhibition of Wnt/β-catenin signaling and ZEB1 expression. Mol Med Rep. (2019) 19:2144–52. 10.3892/mmr.2019.9870 30664209 PMC6390049

[16] Yang P Wu Z Ma C Pan N Wang Y Yan L. Endometrial miR-543 is downregulated during the implantation window in women with endometriosis-related infertility. Reprod Sci. (2019) 26:900–8. 10.1177/1933719118799199 30231774

[17] Dai Y Lin X Xu W Lin X Huang Q Shi L. MiR-210-3p protects endometriotic cells from oxidative stress-induced cell cycle arrest by targeting BARD1. Cell Death Dis. (2019) 10:1–15. 10.1038/s41419-019-1395-6 30760709 PMC6374490

[18] Nabiel Y ELshahawy H Mosbah A. Intrauterine bacterial colonization and endometrial microRNA-17-5p levels in association to endometriosis: a study in an egyptian population. Immunol Investig. (2019). 10.1080/08820139.2019.1693592. [Epub ahead of print]. 31747809

[19] Mashayekhi P Noruzinia M Zeinali S Khodaverdi S. Endometriotic mesenchymal stem cells epigenetic pathogenesis: deregulation of miR-200b, miR-145, and let7b in a functional imbalanced epigenetic disease. Cell J. (2019) 21:179. 10.22074/cellj.2019.5903 30825291 PMC6397607

[20] Chen Y Wang K Xu Y Guo P Hong B Cao Y. Alteration of myeloid-derived suppressor cells, chronic inflammatory cytokines, and exosomal miRNA contribute to the peritoneal immune disorder of patients with endometriosis. Reprod Sci. (2019) 26:1130–8. 10.1177/1933719118808923 30453861

[21] Cosar E Mamillapalli R Moridi I Duleba A Taylor HS. Serum microRNA biomarkers regulated by simvastatin in a primate model of endometriosis. Reprod Sci. (2019) 26:1343–50. 10.1177/1933719118765971 29587611 PMC6949973

[22] Li M Zhou Y Taylor HS. miR-451a inhibition reduces established endometriosis lesions in mice. Reprod Sci. (2019) 26:1506–11. 10.1177/1933719119862050 31354069

[23] Khalaj K Miller JE Lingegowda H Fazleabas AT Young SL Lessey BA. Extracellular vesicles from endometriosis patients are characterized by a unique miRNA-lncRNA signature. JCI Insight. (2019) 4:e128846. 10.1172/jci.insight.128846 31534048 PMC6795291

[24] Maged AM Deeb WS El Amir A Zaki SS El Sawah H Al Mohamady M. Diagnostic accuracy of serum miR-122 and miR-199a in women with endometriosis. Int J Gynecol Obstetr. (2018) 141:14–9. 10.1002/ijgo.12392 29149541

[25] Bashti O Noruzinia M Garshasbi M Abtahi M miR-31 and miR-145 as potential non-invasive regulatory biomarkers in patients with endometriosis. Cell J. (2018) 20:1–131. 10.22074/cellj.2018.4915 29308623 PMC5759684

[26] Cheng F Lu L Wang H Cheng H Zhang D. Expression and significance of miR-126 and miR-145 in infertility due to endometriosis. J Coll Phys Surg Pak. (2019) 29:585–7. 10.29271/jcpsp.2019.06.585 31133163

[27] Marí-Alexandre J Barceló-Molina M Belmonte-López E García-Oms J Estellés A Braza-Boïls A. Micro-RNA profile and proteins in peritoneal fluid from women with endometriosis: their relationship with sterility. Fertil Steril. (2018) 109:675–84. e2. 10.1016/j.fertnstert.2017.11.036 29605406

[28] Pei T Liu C Liu T Xiao L Luo B Tan J. miR-194-3p represses the progesterone receptor and decidualization in eutopic endometrium from women with endometriosis. Endocrinology. (2018) 159:2554–62. 10.1210/en.2018-00374 29762665

[29] Pateisky P Pils D Szabo L Kuessel L Husslein H Schmitz A. hsa-miRNA-154-5p expression in plasma of endometriosis patients is a potential diagnostic marker for the disease. Reprod Biomed Online. (2018) 37:449–66. 10.1016/j.rbmo.2018.05.007 29857988

[30] Rekker K Tasa T Saare M Samuel K Kadastik Ü Karro H. Differentially-expressed miRNAs in ectopic stromal cells contribute to endometriosis development: the plausible role of miR-139-5p and miR-375. Int J Mol Sci. (2018) 19:3789. 10.3390/ijms19123789 30487429 PMC6321240

[31] Zhao L Gu C Ye M Zhang Z Fan W Meng Y. Integration analysis of microRNA and mRNA paired expression profiling identifies deregulated microRNA-transcription factor-gene regulatory networks in ovarian endometriosis. Reprod Biol Endocrinol. (2018) 16:4. 10.1186/s12958-017-0319-5 29357938 PMC5776778

[32] Shu X-M Assessment of the expression of mir-29c, mir-200a and mir-145 in endometrial tissue and the downstream molecules in infertile patients with endometriosis. J Hain Med Univ. (2017) 23:97–100.

[33] Joshi NR Miyadahira EH Afshar Y Jeong J-W Young SL Lessey BA. Progesterone resistance in endometriosis is modulated by the altered expression of microRNA-29c and FKBP4. J Clin Endocrinol Metab. (2017) 102:141–9. 10.1210/jc.2016-2076 27778641 PMC5413101

[34] Nothnick WB Falcone T Joshi N Fazleabas AT Graham A. Serum miR-451a levels are significantly elevated in women with endometriosis and recapitulated in baboons (Papio anubis) with experimentally-induced disease. Reprod Sci. (2017) 24:1195–202. 10.1177/1933719116681519 27920341 PMC6343454

[35] Braicu O-L Budisan L Buiga R Jurj A Achimas-Cadariu P Pop LA. miRNA expression profiling in formalin-fixed paraffin-embedded endometriosis and ovarian cancer samples. OncoTargets Ther. (2017) 10:4225. 10.2147/OTT.S137107 28894379 PMC5584916

[36] Kim MK Lee SK Park JH Lee JH Yun BH Park JH. Ginsenoside Rg3 decreases fibrotic and invasive nature of endometriosis by modulating miRNA-27b: *in vitro* and *in vivo* studies. Sci Rep. (2017) 7:1–14. 10.1038/s41598-017-17956-0 29247225 PMC5732249

[37] Wright KR Mitchell B Santanam N. Redox regulation of microRNAs in endometriosis-associated pain. Redox Biol. (2017) 12:956–66. 10.1016/j.redox.2017.04.037 28499250 PMC5429229

[38] Xu X Li Z Liu J Yu S Wei Z. MicroRNA expression profiling in endometriosis-associated infertility and its relationship with endometrial receptivity evaluated by ultrasound. J X Ray Sci Technol. (2017) 25:523–32. 10.3233/XST-17286 28506024

[39] Yang R Teng H Xu X Liu S Wang Y Guo F. Microarray analysis of microRNA deregulation and angiogenesis-related proteins in endometriosis. Genet Mol Res. (2016) 15:1–8. 10.4238/gmr.15027826 27323121

[40] Xu T Zhao S Dong M Yu X. Hypoxia responsive miR-210 promotes cell survival and autophagy of endometriotic cells in hypoxia. Eur Rev Med Pharmacol Sci. (2016) 20:399–406. 26914112

[41] Falih ES Aubaid SH Yousif WaT The role of microRNA 122, MCP-1 and TGF-β1 as diagnostic biomarkers for endometriosis. Indian J Public Health Res Dev. (2019) 10:2652–6. 10.5958/0976-5506.2019.03267.4

[42] Cosar E Mamillapalli R Ersoy GS Cho S Seifer B Taylor HS. Serum microRNAs as diagnostic markers of endometriosis: a comprehensive array-based analysis. Fertil Steril. (2016) 106:402–9. 10.1016/j.fertnstert.2016.04.013 27179784

[43] Eggers JC Martino V Reinbold R Schäfer SD Kiesel L Starzinski-Powitz A. microRNA miR-200b affects proliferation, invasiveness and stemness of endometriotic cells by targeting ZEB1, ZEB2 and KLF4. Reprod Biomed Online. (2016) 32:434–45. 10.1016/j.rbmo.2015.12.013 26854065

[44] Liu X Bai X Teng Y Song L Lu N Yang R. miRNA-15a-5p regulates VEGFA in endometrial mesenchymal stem cells and contributes to the pathogenesis of endometriosis. Eur Rev Med Pharmacol Sci. (2016) 20:3319–26. 27608888

[45] Hirakawa T Nasu K Abe W Aoyagi Y Okamoto M Kai K. miR-503, a microRNA epigenetically repressed in endometriosis, induces apoptosis and cell-cycle arrest and inhibits cell proliferation, angiogenesis, and contractility of human ovarian endometriotic stromal cells. Human Reprod. (2016) 31:2587–97. 10.1093/humrep/dew217 27619772

[46] Ma Y Huang YX Chen YY. miRNA-34a-5p downregulation of VEGFA in endometrial stem cells contributes to the pathogenesis of endometriosis. Mol Med Rep. (2017) 16:8259–64. 10.3892/mmr.2017.7677 28990049

[47] Seifer BJ Su D Taylor HS. Circulating miRNAs in murine experimental endometriosis: decreased abundance of let-7a. Reprod Sci. (2017) 24:376–81. 10.1177/1933719116667228 27655771

[48] Liang Z Chen Y Zhao Y Xu C Zhang A Zhang Q. miR-200c suppresses endometriosis by targeting MALAT1 *in vitro* and *in vivo*. Stem Cell Res Ther. (2017) 8:251. 10.1186/s13287-017-0706-z 29116025 PMC5678601

[49] Yang W Hong L Xu X Wang Q Huang J Jiang L. Regulation of miR-33b on endometriosis and expression of related factors. Eur Rev Med Pharmacol Sci. (2017) 21:2027−33. 28537685

[50] Chen X Jiang Y Pan D. miR-30c may serve a role in endometriosis by targeting plasminogen activator inhibitor-1. Exp Ther Med. (2017) 14:4846–52. 10.3892/etm.2017.5145 29201189 PMC5704271

[51] Yang W Hong L Xu X Wang Q Huang J Jiang L MiR-424-5p regulates proliferation and apoptosis by targeting FGFR1 in endometriosis cells. Int J Clin Exp Med. (2017) 10:666–74.

[52] Zhang A Wang G Jia L Su T Zhang L. Exosome-mediated microRNA-138 and vascular endothelial growth factor in endometriosis through inflammation and apoptosis via the nuclear factor-κB signaling pathway. Int J Mol Med. (2019) 43:358–70. 10.3892/ijmm.2018.3980 30431056 PMC6257842

[53] Gao S Liu S Gao Z-M Deng P Wang D-B. Reduced microRNA-451 expression in eutopic endometrium contributes to the pathogenesis of endometriosis. World J Clin Cases. (2019) 7:2155. 10.12998/wjcc.v7.i16.2155 31531311 PMC6718782

[54] Zhang M Zhang Y Li L Ma L Zhou C. Dysregulation of miR-202-3p affects migration and invasion of endometrial stromal cells in endometriosis via targeting ROCK1. Reprod Sci. (2020) 27:731–42. 10.1007/s43032-019-00079-4 32046445

[55] Liu Y Lu C Fan L Wang J Li T Liu Z. MiR-199a-5p targets ZEB1 to inhibit the epithelial-mesenchymal transition of ovarian ectopic endometrial stromal cells via PI3K/Akt/mTOR signal pathway *in vitro* and *in vivo*. Reprod Sci. (2020) 27:110–8. 10.1007/s43032-019-00016-5 32046378

[56] Chen L-J Hu B Han Z-Q Ni J Zhou Y-M Chen X-X MicroRNA-20a mediates the cytotoxicity of natural killer cells in endometriosis via ERG/HLX/STAT4/perforin axis. Preprint. (2020). 10.21203/rs.2.17459/v1

[57] Zhang Y Yan J Pan X. miR-141-3p affects apoptosis and migration of endometrial stromal cells by targeting KLF-12. Pflügers Archiv Eur J Physiol. (2019) 471:1055–63. 10.1007/s00424-019-02283-2 31129698

[58] Petracco R Dias ACdO Taylor HS Petracco Á Badalotti M Michelon JdR. Evaluation of miR-135a/b expression in endometriosis lesions. Biomed Rep. (2019) 11:181–7. 10.3892/br.2019.1237 31565224 PMC6759580

[59] Li X Zhang W Fu J Xu Y Gu R Qu R. MicroRNA-451 is downregulated in the follicular fluid of women with endometriosis and influences mouse and human embryonic potential. Reprod Biol Endocrinol. (2019) 17:96. 10.1186/s12958-019-0538-z 31744497 PMC6862852

[60] Ma L Li Z Li W Ai J Chen X. MicroRNA-142-3p suppresses endometriosis by regulating KLF9-mediated autophagy *in vitro* and *in vivo*. RNA Biol. (2019) 16:1733–48. 10.1080/15476286.2019.1657352 31425004 PMC6844572

[61] Kästingschäfer CS Schäfer SD Kiesel L Götte M. miR-142-3p is a novel regulator of cell viability and proinflammatory signalling in endometrial stroma cells. Reprod Biomed Online. (2015) 30:553–6. 10.1016/j.rbmo.2015.01.002 25754227

[62] Zhu H Cao XX Liu J Hua H. MicroRNA-488 inhibits endometrial glandular epithelial cell proliferation, migration, and invasion in endometriosis mice via Wnt by inhibiting FZD7. J Cell Mol Med. (2019) 23:2419–30. 10.1111/jcmm.14078 30729701 PMC6433721

[63] Hu Z Mamillapalli R Taylor HS. Increased circulating miR-370-3p regulates steroidogenic factor 1 in endometriosis. Am J Physiol Endocrinol Metab. (2019) 316:E373–82. 10.1152/ajpendo.00244.2018 30576245 PMC6459299

[64] Meng X Liu J Wang H Chen P Wang D. MicroRNA-126-5p downregulates BCAR3 expression to promote cell migration and invasion in endometriosis. Mol Cell Endocrinol. (2019) 494:110486. 10.1016/j.mce.2019.110486 31233772

[65] Wu D Lu P Mi X Miao J. Exosomal miR-214 from endometrial stromal cells inhibits endometriosis fibrosis. MHR Basic Sci Reprod Med. (2018) 24:357–65. 10.1093/molehr/gay019 29660008

[66] He SZ Li J Bao HC Wang MM Wang XR Huang X. G protein-coupled estrogen receptor/miR-148a/human leukocyte antigen-G signaling pathway mediates cell apoptosis of ovarian endometriosis. Mol Med Rep. (2018) 18:1141–8. 10.3892/mmr.2018.9039 29845209

[67] Hsu CY Hsieh TH Er TK Chen HS Tsai CC Tsai EM. MiR-381 regulates cell motility, growth and colony formation through PIK3CA in endometriosis-associated clear cell and endometrioid ovarian cancer. Oncol Rep. (2018) 40:3734–42. 10.3892/or.2018.6779 30542723

[68] Sahin C Mamillapalli R Yi KW Taylor HS. micro RNA Let-7b: a Novel treatment for endometriosis. J Cell Mol Med. (2018) 22:5346–53. 10.1111/jcmm.13807 30063121 PMC6201226

[69] Park JH Lee SK Kim MK Lee JH Yun BH Park JH. Saponin extracts induced apoptosis of endometrial cells from women with endometriosis through modulation of miR-21-5p. Reprod Sci. (2018) 25:292–301. 10.1177/1933719117711263 28558522

[70] Liu Y Chen J Zhu X Tang L Luo X Shi Y. Role of miR-449b-3p in endometriosis via effects on endometrial stromal cell proliferation and angiogenesis. Mol Med Rep. (2018) 18:3359–65. 10.3892/mmr.2018.9341 30066926 PMC6102748

[71] Wang F Wang H Jin D Zhang Y. Serum miR-17, IL-4, and IL-6 levels for diagnosis of endometriosis. Medicine. (2018) 97:e10853. 10.1097/MD.0000000000010853 29901577 PMC6023682

[72] Sepahi N Kohan L Jahromi AR Daneshbod Y Hoveidi EN. mir-126 rs4636297 and TGF β RI rs334348 functional gene variants are associated with susceptibility to endometriosis and its severity. Gynecol Endocrinol. (2017) 33:429–32. 10.1080/09513590.2017.1290064 28277133

[73] Nimi-Hoveidi E Kohan L Hashemi SS Association of miR-143 rs41291957 and rs4705342 genetic variants with endometriosis risk in infertile women. KAUMS J. (2016) 20:441−6.

[74] Cai H Zhu X Li Z Zhu Y Lang J. lncRNA/mRNA profiling of endometriosis rat uterine tissues during the implantation window. Int J f Mol Med. (2019) 44:2145–60. 10.3892/ijmm.2019.4370 31638262 PMC6844642

[75] Sun P-r Jia S-z Lin H Leng J-H Lang J-H. Genome-wide profiling of long noncoding ribonucleic acid expression patterns in ovarian endometriosis by microarray. Fertil Steril. (2014) 101:1038-46. e7. 10.1016/j.fertnstert.2013.12.035 24502888

[76] Huang H Zhu Z Song Y. Downregulation of lncrna uca1 as a diagnostic and prognostic biomarker for ovarian endometriosis. Rev Assoc Méd Bras. (2019) 65:336–41. 10.1590/1806-9282.65.3.336 30994830

[77] Qiu J-J Lin Y-Y Tang X-Y Ding Y Yi X-F Hua K-Q. Extracellular vesicle-mediated transfer of the lncRNA-TC0101441 promotes endometriosis migration/invasion. Exp Cell Res. (2020) 388:111815. 10.1016/j.yexcr.2020.111815 31911152

[78] Xu Z Zhang L Yu Q Zhang Y Yan L Chen Z-J. The estrogen-regulated lncRNA H19/miR-216a-5p axis alters stromal cell invasion and migration via ACTA2 in endometriosis. Mol Hum Reprod. (2019) 25:550–61. 10.1093/molehr/gaz040 31323679

[79] Liu H Zhang Z Xiong W Zhang L Du Y Liu Y. Long non-coding RNA MALAT 1 mediates hypoxia-induced pro-survival autophagy of endometrial stromal cells in endometriosis. J Cell Mol Med. (2019) 23:439–52. 10.1111/jcmm.13947 30324652 PMC6307811

[80] Zhang C Wu W Zhu H Yu X Zhang Y Ye X. Knockdown of long noncoding RNA CCDC144NL-AS1 attenuates migration and invasion phenotypes in endometrial stromal cells from endometriosis. Biol Reprod. (2019) 100:939–49. 10.1093/biolre/ioy252 30496345

[81] Zhu M Chen L Hu M Shi Z Liu Y. Effects of lncRNA BANCR on endometriosis through ERK/MAPK pathway. Eur Rev Med Pharmacol Sci. (2019) 23:6806–12. 10.26355/eurrev_201908_18719 31486479

[82] Cui D Ma J Liu Y Lin K Jiang X Qu Y. Analysis of long non-coding RNA expression profiles using RNA sequencing in ovarian endometriosis. Gene. (2018) 673:140–8. 10.1016/j.gene.2018.06.046 29920364

[83] Lin D Huang Q Wu R Dai S Huang Z Ren L. Long non-coding RNA AFAP1-AS1 promoting epithelial-mesenchymal transition of endometriosis is correlated with transcription factor ZEB1. Am J Reprod Immunol. (2019) 81:e13074. 10.1111/aji.13074 30506548

[84] Liu J Wang Q Zhang R Zhang C Lin J Huang X. Identification of LINC01279 as a cell cycle-associated long non-coding RNA in endometriosis with GBA analysis. Mol Med Rep. (2018) 18:3850–8. 10.3892/mmr.2018.9387 30106115 PMC6131629

[85] Zhang C Wu W Ye X Ma R Luo J Zhu H. Aberrant expression of CHL1 gene and long non-coding RNA CHL1-AS1, CHL1-AS2 in ovarian endometriosis. Eur J Obstetr Gynecol Reprod Biol. (2019) 236:177–82. 10.1016/j.ejogrb.2019.03.020 30943448

[86] Wang M Hao C Huang X Bao H Qu Q Liu Z. Aberrant expression of lncRNA (HOXA11-AS1) and homeobox A (HOXA9, HOXA10, HOXA11, and HOXA13) genes in infertile women with endometriosis. Reprod Sci. (2018) 25:654–61. 10.1177/1933719117734320 29017417

[87] Wang Y Li Y Yang Z Liu K Wang D. Genome-wide microarray analysis of long non-coding RNAs in eutopic secretory endometrium with endometriosis. Cell Physiol Biochem. (2015) 37:2231–45. 10.1159/000438579 26618670

[88] Liu S-P Tian X Cui H-Y Zhang Q Hua K-Q The messenger RNA and long non-coding RNA expression profiles in ectopic and eutopic endometrium provide novel insights into endometriosis. Reprod Dev Med. (2019) 3:11 10.4103/2096-2924.255992

[89] Qiu J Hua K Exosomal long noncoding RNA-NONHAT076754 faciliates endometriosis invasion and predicts endometriosis recurrence. J Minim Invasive Gynecol. (2019) 26:S12 10.1016/j.jmig.2019.09.044

[90] Qiu J-J Lin X-J Zheng T-T Tang X-Y Zhang Y Hua K-Q. The exosomal long noncoding RNA aHIF is upregulated in serum from patients with endometriosis and promotes angiogenesis in endometriosis. Reprod Sci. (2019) 26:1590–602. 10.1177/1933719119831775 30808247

[91] Li Y Liu Y-D Chen S-L Chen X Ye D-S Zhou X-Y. Down-regulation of long non-coding RNA MALAT1 inhibits granulosa cell proliferation in endometriosis by up-regulating P21 via activation of the ERK/MAPK pathway. MHR Basic Sci Reprod Med. (2019) 25:17–29. 10.1093/molehr/gay045 30371869

[92] Ghazal S McKinnon B Zhou J Mueller M Men Y Yang L. H19 lncRNA alters stromal cell growth via IGF signaling in the endometrium of women with endometriosis. EMBO Mol Med. (2015) 7:996–1003. 10.15252/emmm.201505245 26089099 PMC4551339

[93] Liu Z Liu L Zhong Y Cai M Gao J Tan C. LncRNA H19 over-expression inhibited Th17 cell differentiation to relieve endometriosis through miR-342-3p/IER3 pathway. Cell Bioscience. (2019) 9:84. 10.1186/s13578-019-0346-3 31636893 PMC6792244

[94] Lin K Zhan H Ma J Xu K Wu R Zhou C. Silencing of SRA1 regulates ER expression and attenuates the growth of stromal cells in ovarian endometriosis. Reprod Sci. (2017) 24:836–43. 10.1177/1933719116670036 27694140

[95] Sha L Huang L Luo X Bao J Gao L Pan Q. Long non-coding RNA LINC00261 inhibits cell growth and migration in endometriosis. J Obstetr Gynaecol Res. (2017) 43:1563–9. 10.1111/jog.13427 28707780

[96] Liang Y Wu J Wang W Xie H Yao S. Pro-endometriotic niche in endometriosis. Reprod Biomed Onl. (2019) 38:549–59. 10.1016/j.rbmo.2018.12.025 30772194

[97] García-Bermúdez J Cuezva JM. The ATPase inhibitory factor 1 (IF1): A master regulator of energy metabolism and of cell survival. Biochim Biophys Acta Bioenerget. (2016) 1857:1167–82. 10.1016/j.bbabio.2016.02.004 26876430

[98] Zhang D Jin W Wu R Li J Park S-A Tu E. High glucose intake exacerbates autoimmunity through reactive-oxygen-species-mediated TGF-β cytokine activation. Immunity. (2019) 51:671–81. e5. 10.1016/j.immuni.2019.08.001 31451397 PMC9811990

[99] Kaponis A Iwabe T Taniguchi F Ito M Deura I Decavalas G. The role of NF-kappaB in endometriosis. Front Biosci. (2012) 4:1213–34. 10.2741/s327 22652867

[100] Cho YJ Lee SH Park JW Han M Park MJ Han SJ. Dysfunctional signaling underlying endometriosis: current state of knowledge. J Mol Endocrinol. (2018) 60:R97–113. 10.1530/JME-17-0227 29330150

[101] Hsiao K-Y Wu M-H Tsai S-J Roles of Prostaglandin E 2 in Endometriosis. Endometriosis: Tokyo: Springer (2014). p. 125–46.

[102] Zhang M Li J Duan S Fang Z Tian J Yin H. Comprehensive characterization of endometrial competing endogenous RNA network in infertile women of childbearing age. Aging. (2020) 12:4204–21. 10.18632/aging.102874 32112646 PMC7093184

[103] Park S Lim W Bazer FW Whang K-Y Song G. Quercetin inhibits proliferation of endometriosis regulating cyclin D1 and its target microRNAs *in vitro* and *in vivo*. J Nutr Biochem. (2019) 63:87–100. 10.1016/j.jnutbio.2018.09.024 30359864

[104] Gao W-l Liu M Yang Y Yang H Liao Q Bai Y. The imprinted H19 gene regulates human placental trophoblast cell proliferation via encoding miR-675 that targets Nodal Modulator 1 (NOMO1). RNA Biol. (2012) 9:1002–10. 10.4161/rna.20807 22832245

[105] Keniry A Oxley D Monnier P Kyba M Dandolo L Smits G. The H19 lincRNA is a developmental reservoir of miR-675 that suppresses growth and Igf1r. Nat Cell Biol. (2012) 14:659–65. 10.1038/ncb2521 22684254 PMC3389517

